# Theoretical Analysis Method of Variable Thickness GFRP Tray

**DOI:** 10.3390/ma15072346

**Published:** 2022-03-22

**Authors:** Jianjun Li, Zhaolong Du, Shaobo Geng, Wenmei Han, Yuxuan Wu, Hao Feng

**Affiliations:** 1 School of Science, North University of China, No.3 Xueyuan Road, Taiyuan 030051, China; duzhaolong36@163.com (Z.D.); gengshaobo@nuc.edu.cn (S.G.); taiyuanhanwenmei@126.com (W.H.); fhzbdx123@163.com (H.F.); 2 Curtain Wall Bureaus, Zhejiang Province Institute of Architectural Design and Research, Hangzhou 310000, China; wyxarr@163.com

**Keywords:** GFRP tray, variable thickness, thin plate bending, cavity expansion, theoretical analysis

## Abstract

Glass-fiber reinforced polymer (GFRP) bars are increasingly widely used in slope support instead of steel bars or steel pipes. GFRP Bars are generally connected with the slope by combining conical nut and tray, but the tray stress still lacks corresponding theoretical calculation and strength verification methods. Therefore, assuming that the tray is an equal thickness thin plate, the internal force distribution of the tray is calculated using the thin plate bending and cavity expansion theory, and compared with the finite element numerical analysis results of the tray. The calculation and analysis show that the elastic theoretical solution of internal force distribution of equal thickness tray is basically the same as the numerical simulation solution of variable thickness tray. The tray loading and free surface are controlled by hoop tensile and radial compressive stress, respectively. The inner wall of the free surface of the tray is the weakest part of the tray, and the ultimate strength of a GFRP tray is 35.81–53.00% of the standard tensile strength of Φ20 GFRP bars by distortion energy density. This theoretical method can be used for stress analysis of variable thickness trays and has played technical support for promoting the application of GFRP bars in slope support.

## 1. Introduction

Anchoring technology is widely used in underground engineering support, and materials of anchor solid generally choose reinforcement first. However, in some areas with corrosive solid and groundwater, reinforcement corrosion is increasingly becoming the primary cause affecting the safety and durability of the anchoring system. Due to the behavior of corrosion resistance, suitable cutting, low density, and high tensile strength, glass fiber reinforced polymer (GFRP) bars are first used in the field of mine roadway support instead of reinforcement [1,2,3,4], and then used in slope support [5,6] and floor anti-floating anchorage [7,8,9,10]. To use GFRP bars more reasonably in anchoring technology, domestic and foreign experts and scholars have carried out a large number of experimental and theoretical studies on the material and mechanical properties of GFRP bars, and the results show that GFRP bars can replace reinforcement in slope support anchorage system [11,12,13,14,15,16,17,18,19].

Compared with steel bolts, the GFRP bolt is easy to cut and has no risk. As a support material, it is more environmentally friendly for underground engineering, foundation pit, and slope support, and can solve many disadvantages of traditional steel bolts. However, the top part of a GFRP bolt is often the weakness of anchorage. The tensile strength of a GFRP bar is high, but the shear resistance is poor. The traditional anchorage will produce stress concentration, resulting in the cutting of GFRP bars, and the early failure of anchorage will make the tensile strength of GFRP bars unable to play normally. Therefore, it is very necessary to design reasonable GFRP bar anchorage. After a lot of research and practice, GFRP anchorage has formed bonded anchorage, mechanical clamping anchorage, and bonded and sandwich composite anchorage. The bonded anchorage is formed by filling the adhesive between the steel casing and the GFRP bar. The adhesive is generally made of materials such as pure resin, resin mixed with sand, expansive cement grout, etc. Bonded anchorage are generally suitable for GFRP Bars with a small diameter. The casing size of anchorage is generally long. The bonding material needs to be hardened for a while, and its creep deformation is large. Its durability should be considered when used in acid, alkali, and salt environments [20]. Mechanical clamping anchors include wedge-type anchorage [21], clip-type anchorage [20], and nut-tray anchorage [22,23]. Valter [21] designed a wedge-type anchorage for the GFRP bar anchor. The anchorage performance was studied through test and numerical simulation. It was found that this wedge-type anchorage is suitable for GFRP Bars with partial diameter, and the GFRP Bars with too small diameter are prone to shear failure. The clip in the clip-type anchorage can be made of metal or non-metal. In addition, a soft metal sleeve is often set between the clip and FRP bar to increase the anchoring capacity of the anchor [24]. The disadvantage of clip-type anchorage is that it is easy to produce stress concentration and damage the FRP bar during clamping [25]. Moreover, ACI [26] states that the anchor system is not recommended for testing FRP bars that require more than 400 kN of load to fail the specimen [21].

To overcome the above disadvantages, the literature [22,23] proposed the nut-tray anchorage. The nut-tray anchorage proposed by Sun et al. [23] is separated from each other. The tray is a circular plate and the nut is a straight cylinder. The anchorage of this structure has high requirements for the strength of the nut, and the GFRP nut is easy to break during the anchoring process. Zhang et al. [22] made improvements on this basis. The nut is changed to conical, the tray is changed to variable thickness, and the conical hole in the center of the tray interacts with the conical nut to form an extrusion structure. At present, this kind of structural anchorage is widely used in the GFRP bolt support system in China [27,28,29,30].

The structure of nut-tray anchorage has high requirements for the strength and stiffness of the tray. The existing research shows that the failure of a GFRP anchor mainly occurs at the connection between the tray and the nut, and the failure forms are tension crack of the tray, extrusion failure of the nut, and nut falling off [31]. Jia et al. [32] believe that the tray is cracked or broken mainly because the edge deformation is too large. Large deformation causes tangential tensile failure to break the tray, which extends to the tray center and causes the whole tray to fail. Huang et al. [31] find that the positions at the edge of the tray and both ends of the central hole were more dangerous, and the nut middle is easily damaged by tray extrusion. Gao et al. [33] believe that increasing the stiffener from the lower to the upper end of the small disc can significantly improve the strength of the tray. At this time, the volume increase rate is 3.05%, and the tray bearing capacity increases from 85 kN to 140 kN which increases by 64.7%. Wen [34] uses the ANSYS model to analyze the contact stress and displacement between the GFRP bar and the nut and believes that the total tensile stresses of nuts and bars are transmitted through the contact of the thread tooth surface. The first-three-circle tooth surface bears 70% of the full load, which is minimal after the eighth circle. Although the existing research results have some understanding of the stress mechanism of the GFRP tray, the research results are relatively few, and the stress of the GFRP tray needs to be further analyzed. In the GFRP anchorage system, the tray stress is complex, subject to the transverse load of the supporting rock (soil) body and the extrusion stress of the conical nut. The analysis of the tray in the existing literature is limited to numerical simulation and lacks theoretical analysis. Therefore, this paper tries to explore the simplified calculation method of internal force distribution and ultimate strength of GFRP variable thickness tray in theory.

## 2. Test Methods

### 2.1. Test Materials

#### 2.1.1. GFRP Bar

Φ20 full-threaded GFRP bars produced by a company in Taiyuan, China are used in this experiment, and their parameters are listed in Table 1.

#### 2.1.2. Tray and Conical Nut

**Figure 1:**
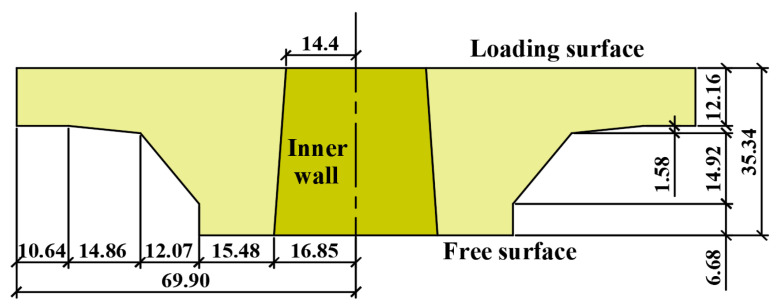
Tray dimensions. Unit in the figure: mm.

Trays and conical nuts used in this experiment are made of GFRP, and their specifications are shown in Figure 1 and Table 2, respectively. One side of the tray is a plane (after this, referred to as the loading surface), which contacts the solid. The other side is a conical surface (after this, referred to as the free surface), in which six stiffeners with a rib width of 10 mm are uniformly distributed outside. The tray center has a conical round hole (the hole wall later referred to as the inner wall), and the larger aperture is located on the free surface.

**Figure 2:**
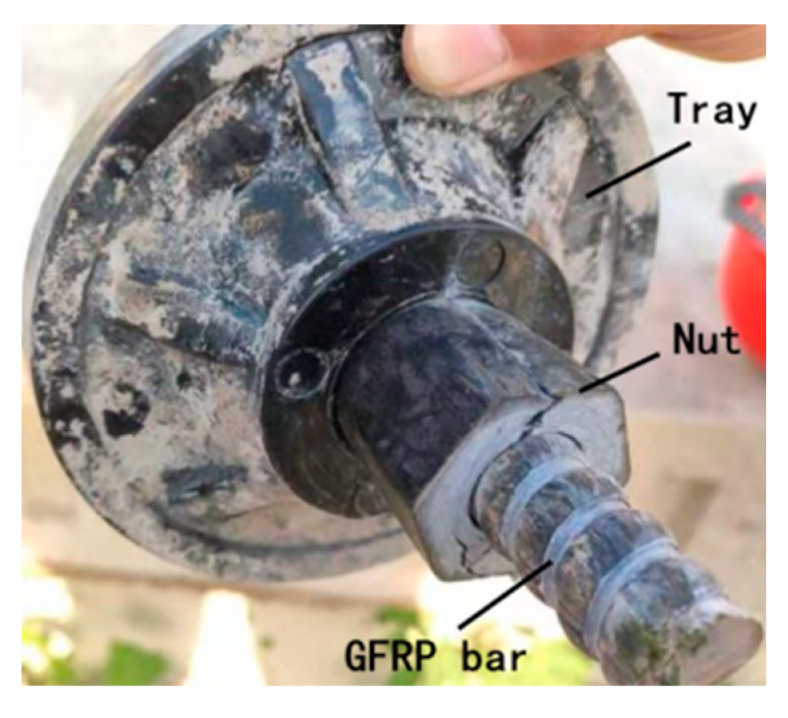
Connection of tray, nut and GFRP bar.

The nut center opens a round hole with the thread arranged on the wall. One end of the outer nut wall is hexagonal and the other is conical, and two 3 mm × 30 mm slits are symmetrically opened on both sides of the vertebral body. The anchor system at the bolt top is formed by passing a GFRP bar through the tray center hole and placing a nut between them. When the tray is stressed, the force is transmitted to the anchor rod body through the nut and then to the soil around the anchor rod body (As shown in Figure 2).

### 2.2. Tensile Strength Test of GFRP Bars

**Figure 3:**
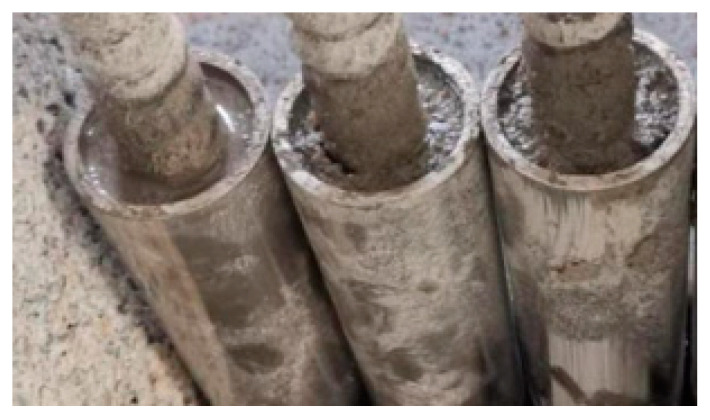
GFRP bars and grouted steel casing.

Three Φ20 GFRP bars with 75 cm long are tested for tensile strength. The ends of each GFRP bar are tied to a steel wire locator and inserted into a 25 cm Φ32 mm × 2 mm steel sleeve tube, then sealed the outer ends of the sleeve tubes. The expansive cement slurry with 4% expansive agent and 0.5 water-cement ratio is injected into the 25 cm high gap between the bar and sleeve tube several times. At the same time, the sleeve tube wall is struck by a iron rod to ensure the slurry is dense and full, as shown in Figure 3. GFRP specimens are cured for 28 days in an environment with a temperature of 20 ± 0.5 °C and humidity of more than 95%. The tensile strength of GFRP bars is tested by WEW-300B hydraulic universal material testing machine (Shanxi Jingchengsheng Engineering Testing Co., Ltd., Thaiyuan, China) with 2 mm/min.

### 2.3. Nut-Tray Anchorage Tests

#### 2.3.1. Anchoring of GFRP Bars

The ultimate bearing capacity test of GFRP trays is completed in the soil nailing wall support project of the Xiyang Utility Tunnel Project. Pre-drill three holes with 110 mm diameter and 6.5 m deep in slope soil, intercept three holes with a length of 7 m Φ20 GFRP bars, install a locator every 1 m along the GFRP bars, put Φ20 GFRP bars into the three holes, and expose 0.5 m, respectively. Then, the cement slurry with a water-cement ratio of 0.6 (raw material is PO 42.5 cement) is injected into holes until filled. A square bearing platform shall be made at the orifice, and the surface of the bearing platform shall be perpendicular to the axis of the GFRP bar. The on-site pull-out test shall be carried out after curing 14 days. 

#### 2.3.2. Test Device

**Figure 4:**
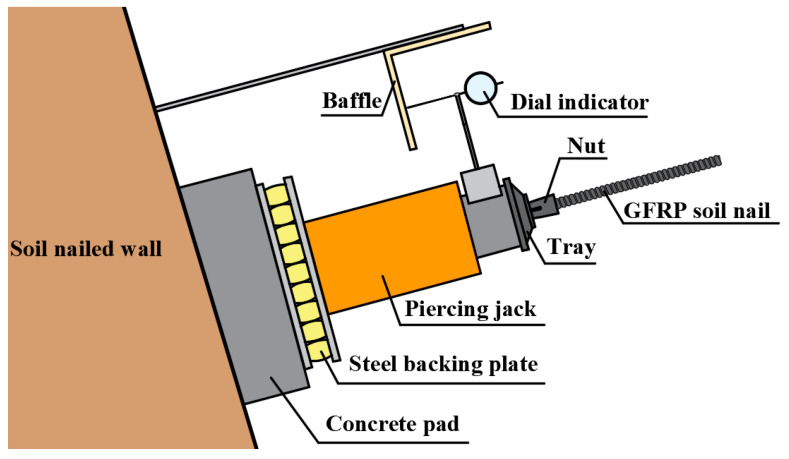
Schematic diagram of field loading test device.

The loading device is arranged according to Figure 4. Test equipment includes a YCW60B50 through-core jack (Shanxi Jiangong Construction Engineering Testing Co., Ltd., Taiyuan, China) with 20 cm stroke, electric oil pump, pressure gauge, digital dial indicator with 0.01 mm accuracy and 30 mm range, and steel pad with rubber pad. The jack, tray, and nut are assembled and debugged after passing through the GFRP bars anchored on the slope in turn. The test loading is carried out to ensure the good working of loading equipment and instruments and a reliable connection between the trays and GFRP bars. After unloading, start the test. 

#### 2.3.3. Test Method

The loading process of this test adopted the multi-cycle loading method. The test process is carried out according to anchor tests in a current specification named ‘Technical specification for retaining and protection of building foundation excavations (JGJ20-2012)’ [35]. Set a load of each level as 6 kN, and record the reading on the dial indicator every 5 min. During the loading process of each level of load, the next level of load can be applied only when the displacement of the anchor head is not greater than 0.1 mm. In case of GFRP bars damage, tray failure, or nut damage during the test, the test shall be stopped.

## 3. Test Results and Analysis

### 3.1. Results Analysis of GFRP Bars Tensile Strength Test

**Figure 5:**
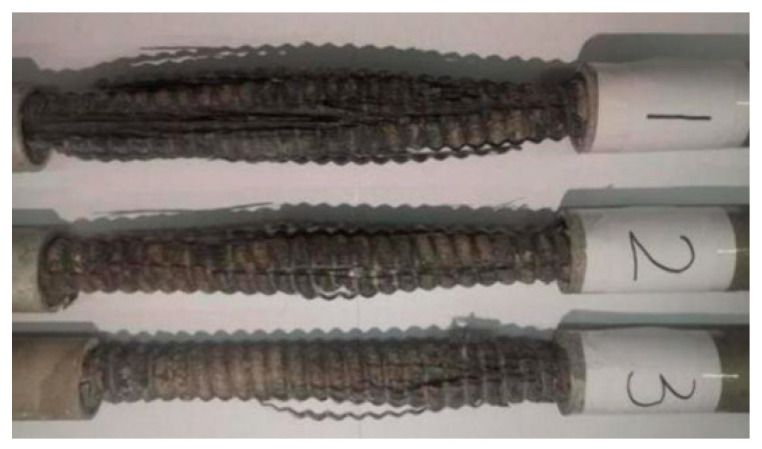
Drawing failure of GFRP bars.

There is no significant change in GFRP bars at the loading beginning in these tests. As the load increase to about $\frac{1}{3}F_{b}$ ($F_{b}$ is the ultimate tensile load of GFRP bars), the specimen emits a slight and intermittent “hissing” sound, and the surface still has no obvious change. When the load reaches $F_{b}$, the specimen makes noise and destroys. The middle part of the specimen gradually uplifts to form a lantern-shaped, and some broken glass fibers are distributed around the uplift in white radial bundles, as shown in Figure 5.

**Figure 6:**
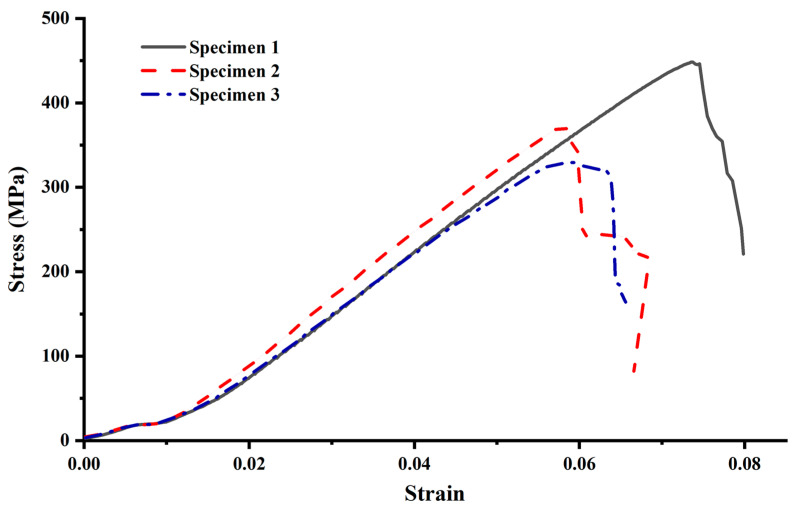
Stress-strain curves of GFRP bars.

The stress-strain curve of GFRP bars consists of three parts: initial gentle section, linear increasing section, and brittle failure section, as shown in Figure 6. The appearance of the initial flat section of the curve may be caused by the slight slip between the specimen chuck and the fixture without preload during the tensile test. As a composite material mainly made of brittle glass fiber, GFRP bars directly change from the elastic stage to the brittle failure stage when reaching the load limit. Due to less glass fiber and more matrix resin, the superficial coat of the GFRP bar has a high slip value and cracks first.

The ultimate tensile strength of three Φ20 GFRP bars is 448.5 MPa, 372.5 MPa, and 353.5 MPa, with an average value of 391.5 MPa. The average elastic modulus of GFRP bars calculated by the linear increase part of the stress-strain curve is 7 GPa, which is quite different from the records of existing literature that will be greater than 40 GPa [6,19]. This difference may be related to the processing technology and glass fiber content of different manufacturers. 

### 3.2. Analysis of Nut-Tray Anchorage Test Results

**Figure 7:**
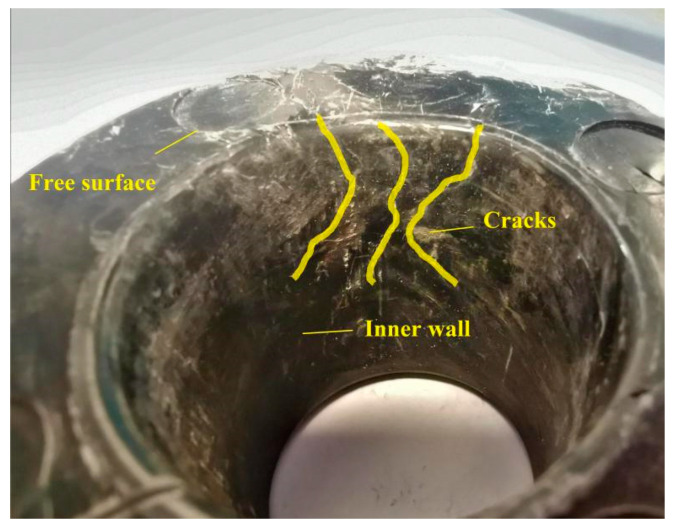
Cracks on the tray.

Three groups of the nut-tray anchorage were tested on-site. In the tests, GFRP bars were not damaged, nuts were cracked to varying degrees, and the relative displacements between nuts and trays were about 10 mm. There is extrusion deformation in the inner wall of the loading surface of the tray. The inner wall of the free surface of the tray has obvious cracks, which are distributed radially along the hole wall, as shown in Figure 7. According to the above phenomena, the failure mode of the nut-tray anchorage test is mainly nut cracking and nut sliding with GFRP bars. The damage of the tray is the local cracking of the inner wall, while the tray surface has no obvious damage and serious deformation, and the deformation of the tray surface is a recoverable small deformation.

The tray maximum loading pressures are 90 kN, 96 kN, and 96 kN, with an average value of 94 kN. Therefore, the tensile strength of GFRP bars in this test is between 286.62 MPa and 305.73 MPa, about 73.21–78.09% of the standard tensile strength (391.5 MPa) of Φ20 GFRP bars and does not exceed 85%. After unloading, the relative displacement between the nut and tray is about 10 mm. In the three tests, one tray produces obvious cracks distributed along radial direction near the free surface on the inner wall, and others have no noticeable damage, as shown in Figure 7.

## 4. Theoretical Analysis on Tray Stress

**Figure 8:**
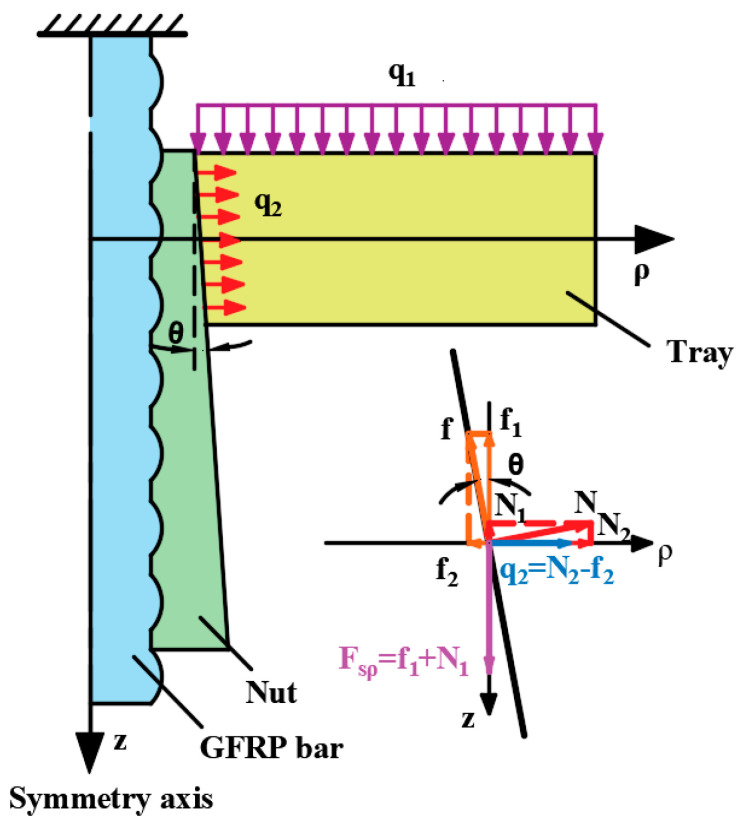
Theoretical calculation model of tray.

In the ultimate bearing capacity test of variable thickness tray, the through center Jack applies a transverse load perpendicular to the loading surface of the tray, and the external force of the tray is transmitted to the GFRP bar through the nut and transformed into the axial load of the GFRP bar. The sidewall of the center hole of the tray forms a circumferential constraint on the nut and bears the extrusion effect caused by the expansion of the nut. In order to theoretically analyze the deformation and internal force of the tray, the variable thickness tray is simplified into an equal thickness circular plate with a central circular hole, and the stress condition of the tray under transverse load is analyzed by thin plate bending theory; The small hole expansion theory is used to analyze the extrusion effect of the nut on the central hole wall of the tray. The internal force and deformation results of the tray can be obtained by superposition of the above two analysis results. Based on the above simplification, it is assumed that the central symmetry axis of the circular plate with equal thickness is the z-axis, and the axis perpendicular to the z-axis along the radial direction of the thickness bisector of the circular plate is defined as ρ-axis, thereby establishing a coordinate system as shown in Figure 8. Through the above analysis, the mechanical analysis of the tray is carried out in the following three steps: first, when calculating the internal force of the tray by using the thin plate bending theory, it is assumed that the tray is integrated with the nut and GFRP bar, and the relative sliding between GFRP bar, nut and tray is not considered; The second step is to simplify the inner wall of the central hole of the tray into an inclined plane, and the included angle between the inclined plane and the z-axis is θ. There are a friction force f parallel to the hole wall and a positive pressure N perpendicular to the hole wall between the nut and the tray hole wall. The friction force f and positive pressure N are decomposed into loads f_1_ and N_1_ parallel to the z-axis, respectively, along the radial direction of the tray ρ-axial loads f_2_ and N_2_. F_sρ_ is the equivalent aperture shear force, which is used to balance the transverse uniformly distributed load acting on the tray. Load q_2_ has an extrusion effect on the hole wall of the tray, and the cavity expansion is used to calculate the internal force of the equal thickness plate; In the third part, the internal force and deformation of the tray are calculated by the above two parts are superimposed to obtain the internal force distribution and deformation of the tray. Thus:

(1) f=Ntanφ

(2) $$F_{s\rho }=N_{1}+f_{1}=N\sin\theta +f\cos\theta$$

(3) $$q_{2}=N_{2}+f_{2}=N\cos\theta -f\sin\theta$$

where φ is the internal friction angle between the nut and the inner wall, tan φ = 0.3. θ is inner wall inclination angle with a value of 0.06922.

### 4.1. Force Analysis of Tray Bending under Transverse Load

The transverse load exerted by the jack on the tray loading surface is simplified as a uniform load, and the contact between the nut and the tray inner wall is facilitated as a fixed bearing. According to the field test results, the tray ultimate failure load is 94 kN, the elastic modulus is 7 GPa, and the Poisson’s ratio is 0.2. The tray can be simplified into two types of equal thickness thin plates. The thickness of thin plates A and B select the tray edge height as 12.16 mm and the tray inner wall height as 35.34 mm, respectively. The inner diameter of the tray is 14.4 mm, and the outer is 69.9 mm. According to the tray loading surface area, the equivalent uniform load $q_{1}$ on the tray loading surface is 6.40 MPa.

According to reference [36], the bending deflection of an axisymmetric circular thin plate can represent Equation (4):

(4) $$\omega =C_{1}\ln\rho +C_{2}\rho^{2}\ln\rho +C_{3}\rho^{2}+C_{4}+\frac{q_{1}}{64D}\rho^{4}$$

where C_1_, C_2_, C_3_, and C_4_ are undetermined coefficients. ω is the bending deflection, m. ρ is the horizontal distance between the tray and the symmetric axis, m. D is bending stiffness, kN·m.

From Equation (4), the equations of rotation, bending moment, and shear force of the tray along the radial direction are

(5) $$\frac{d\omega }{d\rho }=\frac{1}{\rho }C_{1}+2{\rho \ln\rho C}_{2}+{\rho C}_{2}+2{\rho C}_{3}+\frac{q_{1}}{16D}\rho^{3}$$

(6) $$M_{\rho }=-D\left[\frac{\partial^{2}\omega }{\partial \rho^{2}}+\mu \left(\frac{1}{\rho }\frac{\partial \omega }{\partial \rho }+\frac{1}{\rho^{2}}\frac{\partial^{2}\omega }{\partial \varphi^{2}}\right)\right]$$

(7) $$M_{\varphi }=-D\left[\left(\frac{1}{\rho }\frac{\partial \omega }{\partial \rho }+\frac{1}{\rho^{2}}\frac{\partial^{2}\omega }{\partial \varphi^{2}}\right)+\mu \frac{\partial^{2}\omega }{\partial \rho^{2}}\right]$$

(8) $$F_{s\rho }=-D\frac{\partial }{\partial \rho }\left(\frac{\partial^{2}\omega }{\partial \rho^{2}}+\frac{1}{\rho }\frac{\partial \omega }{\partial \rho }+\frac{1}{\rho^{2}}\frac{\partial^{2}\omega }{\partial \varphi^{2}}\right)$$

(9) $$\frac{\partial^{2}\omega }{\partial \rho^{2}}=-\frac{1}{\rho^{2}}C_{1}+2{\ln\rho C}_{2}+3C_{2}+2C_{3}+\frac{3q_{1}}{16D}\rho^{2}$$

(10) $$\frac{\partial^{2}\omega }{\partial \varphi^{2}}=0$$

(11) $$D=\frac{{E\delta }^{3}}{12\left(1-\mu^{2}\right)}$$

where $M_{\rho }$ is the radial bending moment of the tray, kN·m/m. $M_{\varphi }$ is the hoop bending moment of the tray, kN·m/m. $F_{s\rho }$ is the tray radial shear force, kN/m. μ is Poisson’s ratio. E is elastic modulus, MPa. δ is thin plate thickness, m.

The boundary conditions of the tray are:

$${\left(\omega \right)}_{\rho =0.0144}=0,\;{\left(\frac{d\omega }{d\rho }\right)}_{\rho =0.0144}=0$$

$${\left(M_{\rho }\right)}_{\rho =0.0699}=0,\;{\left(F_{S\rho }\right)}_{\rho =0.0699}=0$$

According to the above equations and boundary conditions, the deflection equations of thin plates A and B can be obtained, respectively:

(12) $$\omega_{\delta_{A}}=-2.533\times 10^{-3}\ln\rho -3.575\rho^{2}\ln\rho -7.301\rho^{2}-1.237\times 10^{-2}+91.460\rho^{4}$$

(13) $$\omega_{\delta_{B}}=-1.032\times 10^{-4}\ln\rho -0.1456\rho^{2}\ln\rho -0.2974\rho^{2}-5.040\times 10^{-4}+3.730\rho^{4}$$

**Figure 9:**
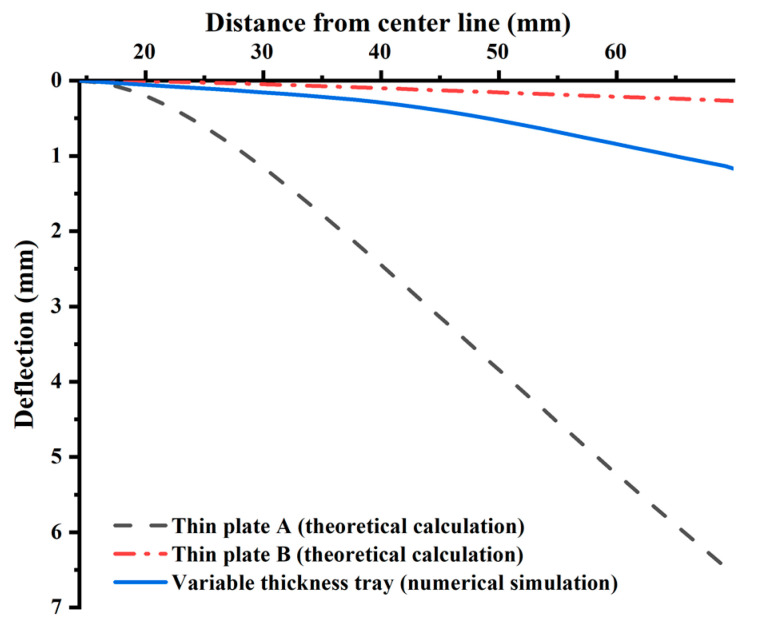
Comparison of radial deflection curves between theoretical analysis and numerical simulation.

The radial deflection curves of the tray are shown in Figure 9. Under the same load conditions and boundary conditions, the tray thickness has a significant influence on the radial deflection amplitude of the tray.

When the thickness is reduced by 2.9 times from 35.34 mm to 12.16 mm, the deflection at the tray edge increases by 24.5 times from 0.30 mm to 7.35 mm.

The radial stress and hoop stress of the tray are:

(14) $$\sigma_{\rho }=\frac{12M_{\rho }z}{\delta^{3}}=-\frac{6M_{\rho }}{\delta^{2}}$$

(15) $$\sigma_{\varphi }=\frac{12M_{\varphi }z}{\delta^{3}}=-\frac{6M_{\varphi }}{\delta^{2}}$$

where $\sigma_{\rho }$ is the radial stress of the tray, MPa. $\sigma_{\varphi }$ is the hoop stress of the tray, MPa.

**Figure 10:**
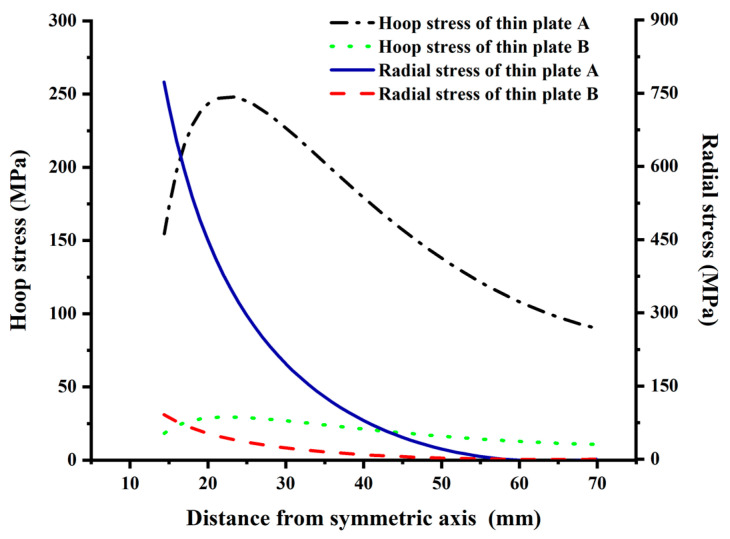
Radial and hoop stress distribution on the loading surface. Note: The radial and hoop stress of the tray free surface are symmetrical to the corresponding stress of the loading surface on the transverse axis, so the diagram is not drawn.

Referring to Figure 10, the radial stress of the tray loading surface is tensile and gradually decreases from the center to the tray edge. At 58 mm away from the symmetrical axis, the radial stresses of thin plates A and B are close to 0. The radial stress attenuation amplitude of thin plate A is faster than B, and the maximum radial stress at the inner wall of thin plate A and B is 773.04 MPa and 91.52 MPa, respectively, with a difference of nearly 8.5 times.

The hoop stress of the free surface of thin plates A and B is tensile stress, and the variation law is the same. The tensile from the center to the edge of the thin plate first increases and then decreases. The difference is that the hoop stress of thin plate A is greater than that of thin plate B. The hoop stresses of thin plate A and B reach the peak at 23 mm from the symmetry axis, which are 248.30 MPa and 29.40 MPa, respectively. The hoop tensile at the inner wall and the edge of thin plates A and B are 154.61 MPa and 18.30 MPa, 90.01 MPa and 10.66 MPa, respectively, nearly 8.4 times larger.

**Figure 11:**
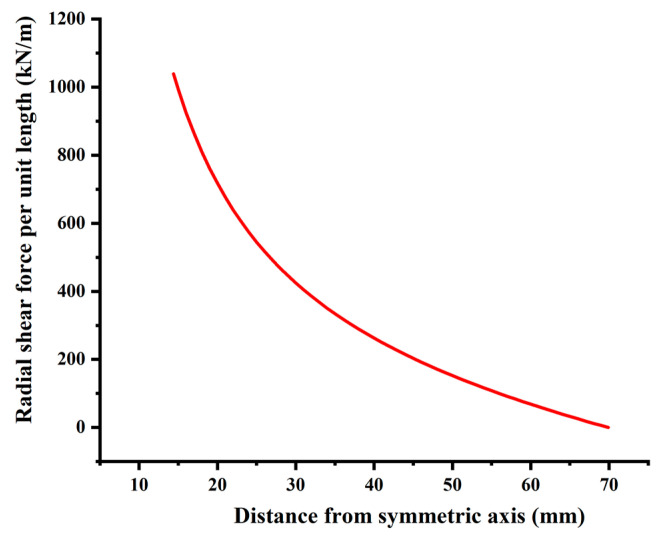
Radial shear force distribution of the thin plate.

Calculate the radial shear $F_{s\rho }$ of the pallet from Equation (8), The results are plotted in Figure 11. The tray thickness does not affect the tray radial shear force. The maximum shear force is 1038.93 kN/m at the inner wall and gradually decreases to 0 along the radial direction.

The resultant shear stress of the tray inner wall $F_{b}=2{\pi \mathrm{rF}}_{s\rho }=2\times \pi \times 0.0144\times 1038.93=94\;\mathrm{kN}$. Equal to the applied external force the theoretical calculation results are reasonable.

In summary, the radial and hoop maximum tensile stress of the thin plate A and B are 773.04 MPa and 248.30 MPa, 91.52 MPa and 29.40 MPa, respectively; thus, the failure is controlled by the radial tensile stress.

### 4.2. Influence of Nut Extrusion on the Tray Internal Force

The extrusion effect of the nut on the inner wall of the tray can be simplified as the cavity expansion problem. From the previous analysis, the squeezing effect on the inner wall of the tray comes from two aspects: the radial component generated by the positive pressure perpendicular to the inner wall of the tray and the radial component caused by the friction parallel to the inner wall of the tray. The resultant force of these two components jointly produces radial expansion on the tray, as shown in Figure 8.

There is friction contact between the nut and the tray inner wall. Both the nut and the tray are GFRP composites, whose friction coefficient is mainly related to the matrix material, glass fiber content, and stress conditions. According to the existing literature [37,38,39,40], although affected by glass fiber content and test conditions, the friction coefficient has been between 0.27 and 0.34. Therefore, in the analysis, the friction coefficient between the nut and the tray can be selected as 0.30.

In the calculation, the tray inner radius is determined according to the equivalent aperture, which is selected as the average value $\rho_{1}$ = 15.625 mm. The hole height (h) is determined according to the actual height of the tray inner wall, h = 35.34 mm. According to the unfavorable factors, the influence range of small hole expansion is the radius of the small circular surface of the tray, $\rho_{2}$ = 32.33 mm. Therefore, from Equation (1) to Equation (3), the radial stress $q_{2}$ acted on the equivalent radius inner wall can be calculated as 71.83 MPa.

**Figure 12:**
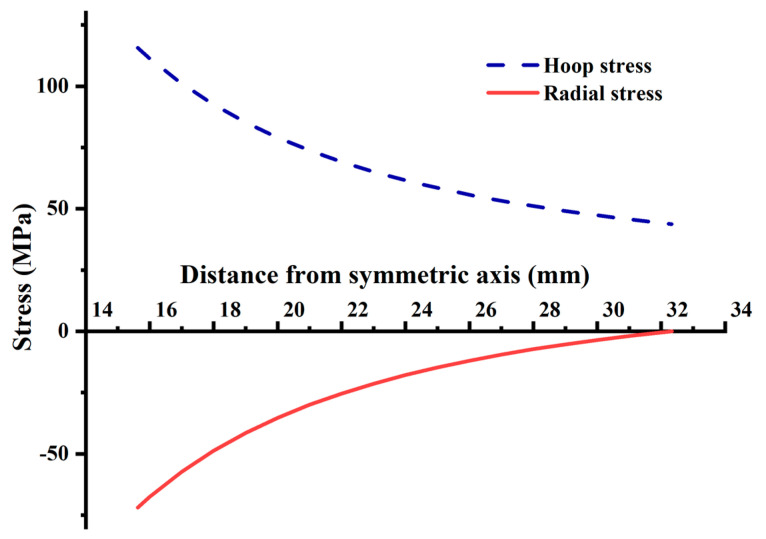
Stress distribution of the tray caused by small hole expansion.

The tray stress under extrusion is calculated according to Equations (16) and (17), and its distribution is shown in Figure 12:

(16) $$\sigma_{\rho }=-\frac{\frac{\rho_{2}^{2}}{\rho^{2}}-1}{\frac{\rho_{2}^{2}}{\rho_{1}^{2}}-1}q_{2}$$

(17) $$\sigma_{\varphi }=\frac{\frac{\rho_{2}^{2}}{\rho^{2}}+1}{\frac{\rho_{2}^{2}}{\rho_{1}^{2}}-1}q_{2}$$

where $\rho_{1}$ is the tray equivalent inner diameter, m. $\rho_{2}$ is the maximum equivalent radius of the cavity expansion effect, m. ρ is the distance between the calculated point and the tray symmetrical axis, m. $\sigma_{\varphi }$ is the hoop stress produced by the cavity expansion, MPa. $\sigma_{\rho }$ is the radial stress created by the cavity expansion, MPa. $q_{2}$ is the radial load applied to the tray inner wall, MPa.

The radial and hoop stress caused by the nut extrusion on the tray is compressive and tensile, respectively, as shown in Figure 12. The maximum hoop and radial stresses are 115.62 MPa and 71.83 MPa, respectively, which are at the tray inner wall, and then gradually decrease along the tray radial direction. The hoop stress plays a leading role along the tray radial direction and is larger than the radial stress. At the equivalent radius of 32.33 mm, the radial stress is 0, and the hoop tensile is 43.78 MPa.

### 4.3. Superposition of Tray Internal Force

From the previous analysis, the internal force distribution of the tray reflects the extrusion effect of the nut on the inner wall of the tray and the bending effect of the tray surface under the transverse uniformly distributed load. Therefore, the internal forces generated by them should be superimposed during radial and hoop stresses of the tray are calculated by theory. According to the cavity expansion results, the stresses produced by nut extrusion on the tray are radial compressive and hoop tensile stress. For transverse load, the radial and hoop stresses of the tray loading and free surface are tensile and compressive stress, respectively. Stresses on two surfaces are mirror symmetry according to the transverse axis. The results of superposition of thin plate bending and small hole expansion are as follows: for the stress surface, the hoop tensile stress increases and the radial stress decreases; For the free surface, on the contrary, the hoop stress decreases, and the radial stress increases.

**Figure 13:**
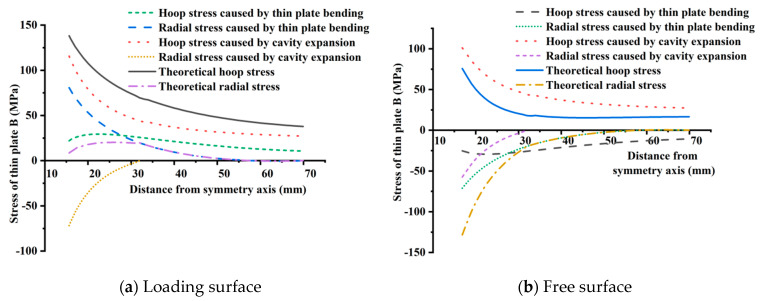
The stress superposition of the loading and free surface of the thin plate B.

The tray stress superposition analysis of radial and hoop stresses is carried out with the thin plate B as an example, as shown in Figure 13a. For the radial stress of the tray loading surface, on the one hand, the radial compressive stress caused by the cavity expansion becomes 0 at the distance of 32.33 mm from the tray symmetry axis, and its influence range is small and numerical attenuation is fast. After superimposing the radial tensile stress caused by thin plate bending and the radial compressive stress caused by cavity expansion, the resultant radial stress is tensile. On the other hand, although the radial compressive stress caused by cavity expansion has a minor influence range, its stress value near the tray inner wall is almost close to the radial tensile stress caused by the thin plate bending. Therefore, the superposition makes the radial stress smaller, which is beneficial to the tray inner wall. The trend of resultant radial stress increases first and then decreases. The pressure at the tray inner wall has a significant influence, which is 8.51 MPa. The resultant maximum radial stress is only 20.27 MPa.

For the hoop stress of the loading surface of the tray, the stresses generated by the thin plate bending and the cavity expansion are all tensile. The resultant hoop stress increase significantly, its trend decreases from the tray inner wall to the outside, and the tray equivalent inner wall (15.625 mm from the symmetrical axis) has the largest stress (138.09 MPa). Within the range of stress superposition, the contribution rate of transverse load in the resultant hoop tensile stress after superposition is 16~37%, and the contribution rate of cavity expansion is 8~63%. Among them, the difference in the contribution rate at the inner wall of the tray is the largest. The contribution rates of cavity expansion and transverse load are 84% and 16%, respectively. Cavity expansion plays a leading role in the hoop stress of the tray and cannot be ignored.

The same method is used to analyze the free surface combined stress of the thin plate B. The radial stresses produced by the thin plate bending and the cavity expansion are all compressive stress, and the resultant radial stress decreases from the tray inner wall to the outside. The tray inner wall (16.85 mm from the symmetrical axis) has the maximum radial stress of 128.37 MPa. The proportion of the stresses produced by the thin plate bending and the cavity expansion is about 55% and 45%, respectively. The properties of hoop stress caused by transverse load and cavity expansion are just opposite, one is compressive stress and the other is tensile stress. The compressive stress generated by thin plate bending has a weakening effect on the tensile stress caused by cavity expansion, which is reduced by about 33 %. The resultant hoop stress of the free surface is tensile stress, which decreases from the tray inner wall to the outside. The maximum stress appears in the tray inner wall (16.85 mm from symmetrical axis, 75.77 MPa). The radial compressive stress of the tray free surface is much larger than the hoop tensile stress, about 1.69 times, so the radial stress controls the free surface, as shown in Figure 13b.

In summary, the cavity expansion effect is significant on the loading surface and free surface of the tray, so it cannot be ignored. The hoop tensile stress of the loading surface is greater than the radial tensile stress, and the radial compressive stress of the free surface is greater than the hoop tensile stress. Therefore, the loading surface of the thin plate B is controlled by the hoop tensile stress. On the contrary, the free surface of the thin plate B is controlled by the radial compressive stress controls. Comparison from the numerical absolute value, the hoop stress on the loading surface of thin plate B is higher than the radial stress on the free surface. We can also draw a similar conclusion for thin plate A.

## 5. Tray Force Analysis of the Numerical Simulation

GFRP tray has a thick middle and thin edge, which is not an ideal thin plate strictly. To analyze the tray limit state stress, Ansys Workbench 2020 R2 software is used to simulate the deformation and internal force of the tray under ultimate transverse load. Firstly, the theoretical calculation and numerical simulation of thin plates A and B under transverse load are compared to verify the rationality of the tray parameter selection. Then according to the geometric size of Figure 1, the axisymmetric numerical model is established to simulate the tray.

The previous Section 3.2 show that under the maximum load of the test, the inner wall of the tray appears cracks, and the tray surface is not obviously damaged. The deformation of the tray surface is recoverable. It can be considered that the tray is still in the elastic stage under the maximum load. Therefore, the linear static analysis is used in the numerical simulation of the tray force in this section. In the numerical simulation, the elastic modulus was measured by Section 3.1, that is, the elastic modulus is 7.0 GPa. The parameters of the tray, nut, and GFRP bar, such as the geometric size, the spatial contact relationship, the boundary conditions, and the applied load, are the same as those of Section 3.2.

### 5.1. Rationality Verification of the Numerical Simulation Parameters

**Figure 14:**
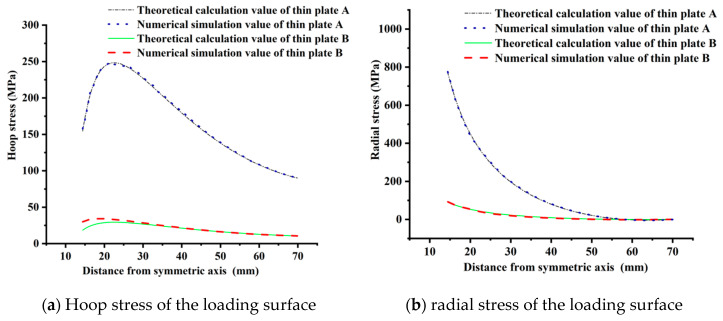
Comparison of theoretical and simulation values of thin plate bending.

The calculation results of the loading surface of thin plates A and B are shown in Figure 14. The numerical simulation of radial and hoop stress of thin plates A and B are consistent with the theoretical calculation, which is different 0.48~1.77%. Therefore, the numerical model element set and the material parameter value are appropriate.

### 5.2. Numerical Simulation Results of the Tray

The numerical model carries out solid 3D modeling according to the actual size (as shown in Figure 1) and contact relationship of the tray. A nut is set between the tray and GFRP bar, and a thread is set between the nut and GFRP reinforcement. The friction contact element is set between the tray and the nut, in which the contact stiffness is 1 and the friction coefficient is 0.3. The element of the numerical model adopts tetrahedral solid element solid187. The numerical model is divided into 84548 grid elements, and the places with large stress and sudden change are locally densified. The density is 2.0 g/mm and Poisson’s ratio is 0.2. The stress-strain relationship adopts the test results of the GFRP pull-out test, and the elastic modulus is 7 GPa. The boundary condition is that one end of the GFRP bar is fixed displacement and the other end is a free boundary. The loading method adopts one-time loading, and the uniformly distributed load is applied on the loading surface of the tray, with the size of 6.40 MPa. The simulation time is 195.19 s.

The tray radial deflection curve obtained by numerical simulation is shown in the natural line in Figure 9, between the theory deflection of the thin plate A and B. The tray deflection increases gradually from the inner wall to the outside, and the outer edge has the maximum deflection (1.17 mm). The tray edge simulation value is 0.16 and 3.9 times the theoretical values of the thin plate A (0.30 mm) and B (7.35 mm). Therefore, the deflection simulation value of the tray is closer to the thin plate B along the radial direction. The initial section of the slope of the tray deflection simulation value is small, which increases significantly between 35 mm and 50 mm from the symmetry axis. The variation range of the deflection simulation value slope is basically consistent with the range of the tray thickness thinning area (32.33–44.40 mm). Therefore, the variation of the tray radial thickness has a significant impact on the radial deflection.

**Figure 15:**
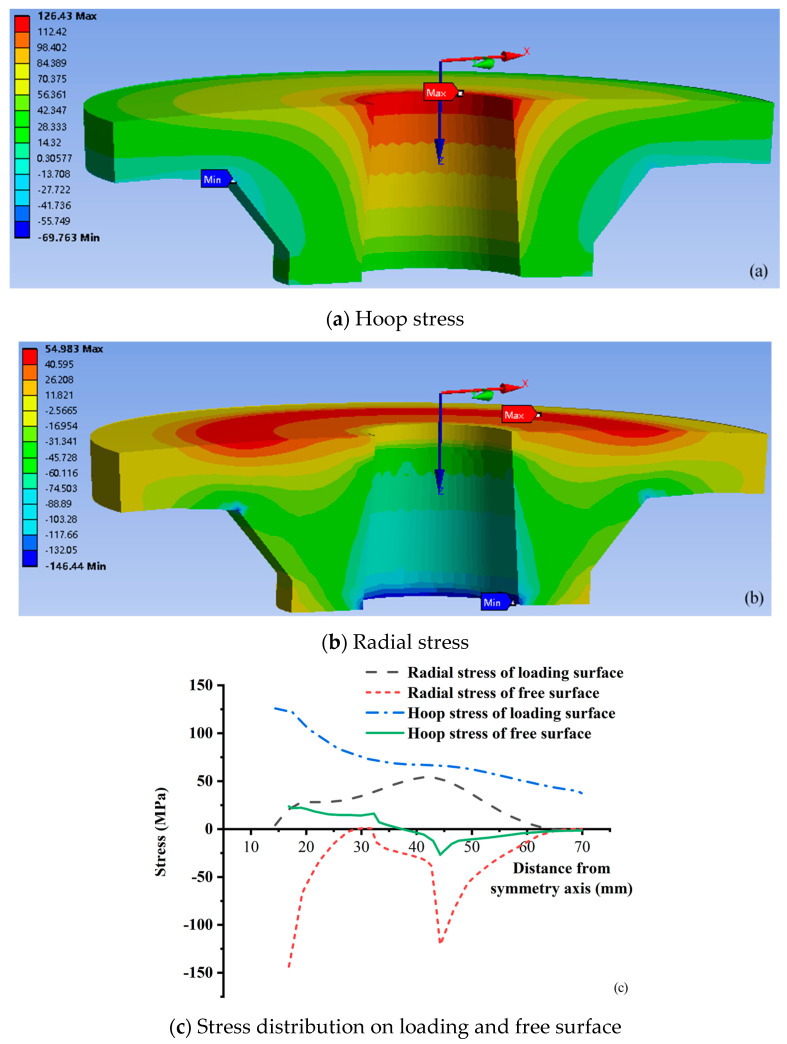
Numerical simulation stress distribution of the tray.

The numerical simulation results of the radial stress and hoop stress of the tray are shown in Figure 15a,b, and the radial stress and hoop stress of the corresponding loading surface and free surface are shown in Figure 15c. It can be seen from Figure 15 that the hoop stress on the loading surface is mainly tensile. From the inner wall (14.4 mm away from the symmetric axis) to the edge of the tray, the hoop stress gradually decreases, and the maximum tensile of the inner wall is 126.43 MPa.

The radial stress of the tray loading surface is mainly tensile and becomes compressive stress after about 62 mm from the symmetric axis, which value increases first and then decreases. The maximum tensile stress (54.983 MPa) is near 42 mm from the symmetry axis, and the maximum compressive stress (1.24 MPa) is near the outer tray edge. Radial compressive stress dominates in the free surface and has a bimodal curve. The first stress peak (146.44 MPa) is at the tray inner wall (16.85 mm away from the symmetric axis), and the second (120.57 MPa) is at 44.40 mm away from the symmetric axis. From the tray inner wall to the outside, the hoop stress of the free surface changes from tensile stress to compressive and then to tensile. The maximum tensile stress at the center hole wall of the tray is 23.67 MPa, and the maximum compressive stress at 44.40 mm away from the symmetry axis is 26.81 MPa. The maximum stress of the tray is the radial compressive stress at the junction of the inner wall and the free surface, with the size of 146.44 MPa. The maximum stress is consistent with the location of the tray crack, as shown in Figure 7. Figure 1 shows that 44.40 mm away from the symmetry axis is the thickness turning area of the tray free surface from thick to thin (after this called thickness turning zone, TTZ), and thus the tray center hole and the thickness turning area has an important influence on the tray stress distribution. As known in the stress envelope, respectively, taking the hoop tensile and radial compressive stress distribution line of the loading and free surface as the boundary, the tray stress analysis can control all unfavorable factors.

**Figure 16:**
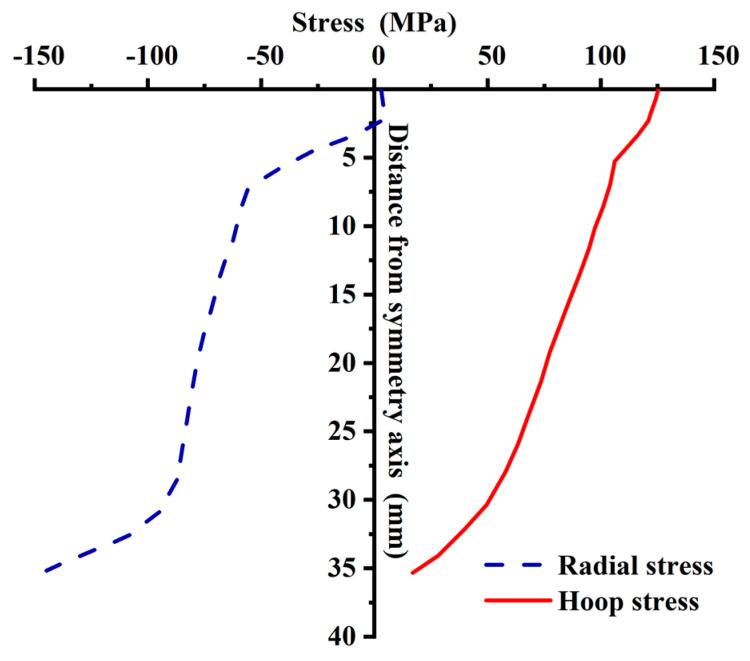
The stress distribution of the inner wall of the tray by numerical simulation.

The radial and hoop stresses of the tray inner wall are shown in Figure 16. The hoop and radial stresses of the tray inner wall are tensile and compressive stress, respectively, which are consistent with the theoretical analysis result of the cavity expansion. The distribution of hoop tensile and radial compressive stress is opposite. From the loading surface of the tray to the free surface, the hoop tensile stress decreases from 126.43 MPa to 23.67 MPa, 85.44 MPa. The radial stress is generally compressive, and the local tensile stress is near the loading surface. The maximum tensile stress is 4.14 MPa, and the maximum compressive stress is 146.44 MPa near the free surface, with an average of 67.62 MPa. Compared with the theoretical calculation of cavity expansion, the hoop tensile stress theoretical value of the inner wall is 115.51 MPa, and the error is 26.03%; The theoretical value of the radial compressive stress of the inner wall is 71.83 MPa, and the error is 5.87%. The theoretical solution of the radial stress on the tray inner wall is close to the numerical simulation results, however, the hoop stress isn’t. The reasons are the following two points. (1) The center hole and the radial thickness of the tray are changed in the numerical simulation but use the equivalent radius in the theoretical calculation. Therefore, there is a particular deviation. (2) The influence of the wall thickness on the hoop tensile stress is more considerable than radial compressive stress. When the distance from the inner wall is close to the hole wall thickness, the radial stress tends to 0. however, the hoop stress is still considerable. The wall thickness is changed in the numerical analysis of the tray. Compared with the wall thickness of the theoretical calculation, the wall thickness of the loading surface changes significantly, and that of the free surface changes little. Therefore, the hoop stress deviation is relatively large while the radial is relatively small.

## 6. Discussion

### 6.1. Comparison of Theoretical Calculation and Numerical Simulation

The similarities and differences between theoretical analysis and numerical simulation in terms of geometric parameters, physical parameters, boundary conditions, load type and size, analysis method, and calculation results are shown in Table 3. The elastic modulus, Poisson’s ratio, and friction coefficient of the two analysis methods are the same, the boundary conditions and load types and sizes are the same, and the analysis methods are linear elasticity. However, the geometric parameters of the tray used in the two analysis methods are quite different. The geometric model used in the numerical simulation is modeled according to the actual size of the tray. The radial thickness of the tray is variable thickness, and the central opening is conical. In order to adopt elastic mechanics analysis, the thickness of the tray is simplified to equal thickness in theoretical analysis, and the central opening is simplified to an equal diameter circular hole.

**Figure 17:**
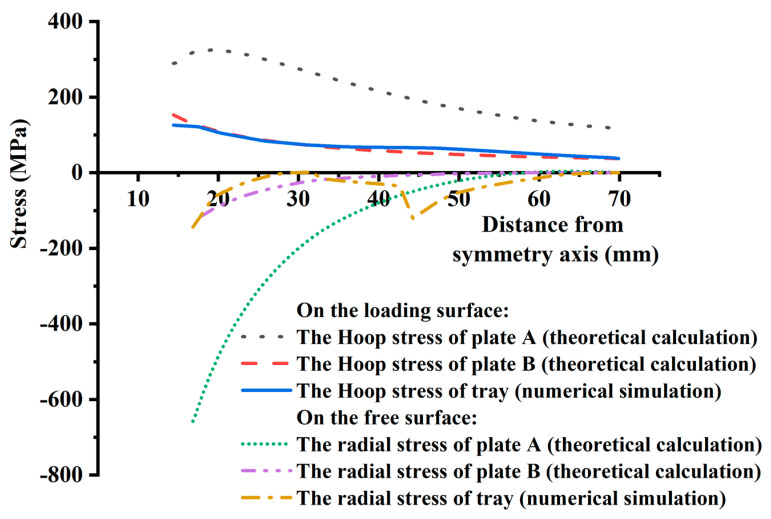
Comparison of stress distribution between theoretical analysis and numerical simulation of the tray.

Both theoretical calculation and numerical simulation show that the failure is controlled by the most unfavorable factors, in which the loading surface of the tray is controlled by hoop stress and the free surface is controlled by radial stress, as shown in Figure 17. For the hoop stress of the loading surface, the tray numerical simulation result is close to the theoretical calculation result of the thin plate B. As for thin plate B, the average value of the theoretical calculation is 64.62 MPa and slightly smaller than the average value (75.35 MPa) of the numerical simulation by 14.24%. The average value of theoretical calculation of thin plate A is 214.91 MPa, which is 185.21% higher than the average value of numerical simulation. For example, the simulated hoop stress of the loading surface is about 124.65 MPa at the tray equivalent inner wall (15.625 mm away from the symmetrical axis), and the theoretical hoop stresses of thin plate A and B are 305.41 MPa and 138.09 MPa, respectively, which are 145.01% and 10.78% higher than the simulated values.

For the tray free surface, the stress concentration occurs at TTZ in the numerical simulation results, which leads to peak radial compressive stress and the bimodal curve of the compressive stress of the free surface. The theoretical calculation results of the thin plates A and B are all the maximum values at the tray inner wall, which causes the difference between the theoretical calculation and the numerical simulation curves of the radial stress of the free surface. For thin plate A, the radial stress (657.69 MPa) at the tray inner wall (16.85 mm away from the symmetrical axis) is far more than the ultimate tensile strength (391.5 MPa) of GFRP bars measured by the tensile strength test. Therefore, the theoretical calculation ultimate tensile strength of thin plate A is quite different from the test results, that is, the thickness assumption of thin plate A is unreasonable.

For thin plate B, the simulated and theoretical values of radial stress on the free surface at the inner wall of the tray are 146.44 MPa and 128.37 MPa, respectively. The theoretical calculation of thin plate B is 12.34% less than the simulated value. At the tray TTZ, the numerical simulation value (120.57 MPa) of radial stress is pretty different from the theoretical calculation value (5.69 MPa). Therefore, it is suggested that measures should be taken to reduce local stress concentration in the tray design. By comparing the hoop stress of the loading surface and the radial stress of the free surface of the tray, their numerical simulation values are close to the theoretical calculation value of the thin plate B. For the hoop tensile stress of the loading surface, the numerical simulation result is consistent with the theoretical calculation result in the numerical value and the variation law along the radial direction. Without considering the abrupt change of radial compressive stress caused by the drastic shift in tray thickness, at the tray inner wall of thin plate B, the numerical simulation and the theoretical calculation values of radial compressive stress have similar values and distribution laws. Therefore, for the variable thickness tray of GFRP, the tray is simplified into a thin plate with equal thickness according to the thickness of the central opening, and the internal force superposition analysis is carried out according to the axisymmetric thin plate bending theory and cavity expansion theory of elasticity, which can theoretically predict the internal force distribution and the main controlling factors of failure of the tray.

It can be seen that the radial deflection, radial stress, and hoop stress of equal thickness plate B calculated by the theoretical analysis have the same variation as the numerical simulation results, and the numerical values are also consistent. The local difference between the theoretical analysis and the numerical simulation results is mainly caused by the difference in the geometric size of the tray.

### 6.2. Ultimate Strength of Tray Failure

According to the tray ultimate bearing capacity test, the maximum loading average value of the tray is 94 kN, which is converted to the GFRP bar strength of 299.22 MPa. The loading of the ultimate bearing capacity test is equivalent to 76.43% of the GFRP bar standard value (391.5 MPa) of the ultimate tensile strength. Considering the discreteness and brittle failure of the tensile strength test value of the GFRP bar, according to the most unfavorable consideration, 84.64% of the minimum test value (353.5 MPa) has been loaded in the tensile strength test, which meets the maximum loading requirement of 85% not exceeding the standard value of the tensile strength specified in the specification JGJ120-2012.

The theoretical calculation and numerical simulation results show that the inner wall and the TTZ of the tray loading and free surface are the places where the stress is enormous. Therefore, the theoretical equation of the distortion energy density, namely Equation (18), can be used to calculate the material strength:

(18) $$\sigma_{t}=\sqrt{0.5\left({\left(\sigma_{1}-\sigma_{2}\right)}^{2}+{\left(\sigma_{2}-\sigma_{3}\right)}^{2}+{\left(\sigma_{3}-\sigma_{1}\right)}^{2}\right)}$$

where $\sigma_{1}$, $\sigma_{2}$ and $\sigma_{3}$ are the maximum, intermediate, and minimum principal stresses, respectively, MPa.

Calculation results of tray strength are shown in Table 4.

Table 4 shows that the tray strength calculated by the theoretical calculation and numerical simulation according to the distortion energy density theory is between 140.18 MPa and 207.50 MPa, of which the strength calculated by theoretical calculation and numerical simulation of the thin plate B at the loading face inner wall are 171.74 MPa and 154.21 MPa, respectively, with a difference of about 10.21%; The strength at the free face inner wall is 207.50 MPa and 190.98 MPa, respectively, and the difference is only about 7.69%. At only 35.81% to 53.00% (average proportion is 44.17%) of 391.5 MPa, these strengths are far less than the Φ20 GFRP bar standard value of ultimate tensile strength. However, the on-site test of the tray shows that there are light cracks on the surface of the tray, which proves that the tray has reached the limit state. Therefore, it is speculated that the ultimate strength of the tray may be inconsistent with that of the GFRP bars material, and the ultimate strength of the tray should be less than the uniaxial tensile strength of the GFRP bars material. The reasons are as follows: (1) The glass fiber distribution in the tray is different from GFRP bars. The glass fiber can be approximately unidirectional in GFRP bars but at least two-dimensional distribution in the tray. (2) The strength difference is caused by the inconsistency of glass fiber distribution direction and stress direction. For GFRP fiber composites, the tensile force is mainly borne by glass fiber. GFRP bars are subjected to a unidirectional tensile force, which has the same orientation as the glass fiber of GFRP bars. Therefore, the ultimate tensile strength of glass fiber can be fully played. The tray is stressed in both radial and circumferential directions. One dimensional glass fiber can not meet the stress in two directions at the same time, resulting in that only some fibers bear tensile force in each stress direction. Assuming that the glass fiber content of a tray and GFRP bar is the same, the strength of the tray under two-way stress is at least 50% lower than that of the GFRP bar under one-way stress. Accordingly, the tray strengths calculated by the distortion energy density theory and numerical simulation have reached the tray ultimate bearing capacity. However, they are about 50% of the Φ20 GFRP bar standard value of the ultimate tensile strength. The rationality that the calculated tray strength is about 50% lower than the ultimate tensile strength of GFRP bars further proves the rationality of using the theoretical calculation result of thin plate B. The distortion energy density theory considers the force and deformation when calculating the tray strength. Therefore, it is feasible and reasonable for the tray made of GFRP reinforced composites to use the distortion energy density theory to calculate the strength.

### 6.3. Potential Strategy to Prevent Failure of Trays

Based on the theoretical analysis and numerical simulation results of the above tray stress distribution and ultimate strength, the following improvement measures are proposed:

(1) Increase the glass fiber content of the tray, and improve the strength and deformation resistance of the tray from the perspective of materials.
(2) Optimize the appearance shape of the free surface of the tray and reduce the stress concentration caused by the sudden change of the thickness of the tray, so as to improve the stiffness of the tray and reduce the radial deformation of the stressed surface.
(3) Increase the width of the small circular surface of the free surface of the tray and improve the radial restraint ability of the central orifice of the tray free surface to the nut.
(4) Optimize the cone angle of the nut and reasonably distribute the extrusion and shear capacity of the nut.

## 7. Conclusions

A stress analysis method of variable thickness tray is proposed. In this method, the variable thickness tray is simplified into an equal thickness thin plate. The stress distribution of the tray is calculated by superposition of thin plate bending theory and cavity expansion theory, and the calculation method of the tray ultimate strength is discussed.

In the nut-tray anchorage test, the failure modes are mainly nut cracking and nut sliding with GFRP bars. The tray only appears local cracking damage in the inner wall of the free surface, but the tray surface has no obvious damage, and the deformation of the tray surface is recoverable. Therefore, the elastic analysis is used in the theoretical analysis, and the linear static analysis is used in the numerical simulation. The disk load is the same as the nut-tray anchorage test, and the elastic modulus is tested by GFRP bar tensile strength test.

The thickness of the tray is the main factor affecting the size and spatial distribution of the radial deflection, radial stress, and hoop stress of the tray. In the simplified analysis, the reasonable selection of the equivalent thickness of the variable thickness tray is the key to solving the problem. By comparing the theoretical calculation and numerical simulation results, it is considered that the variable thickness tray is simplified to the equal thickness tray according to the thickness of the hole wall of the central opening of a tray, and the calculation results of tray internal force are reasonable and feasible.

The radial stress and hoop stress of the loading surface and free surface of the tray are the results of the superposition of the transverse load acting on the stressed surface of the tray and the extrusion effect of the nut on the inner wall. The stress superposition causes the tray loading surface is controlled by hoop tensile stress, and the free surface is controlled by radial compressive stress. In terms of numerical value, the hoop tensile stress on the loading surface of the tray is much greater than the radial compressive stress on the free surface.

On the loading surface of the tray, the contribution of nut extrusion to the hoop stress is dominant, accounting for 84~63%; The contribution of tray lateral load to the hoop stress occupies a secondary position, accounting for 16~37%. From the central hole wall of the tray to the edge of the tray, the hoop stress caused by the extrusion of the nut gradually decreases from large to small.

The extrusion of the nut causes the stress distribution of the central hole wall of the tray to change greatly along the thickness direction. From the loading surface of the tray to the free surface, the hoop tensile stress is dominant, and gradually transits to the radial compressive stress. The difference in the distribution of glass fiber may be the main reason for the difference in the ultimate strength between the tray and the GFRP bar. For the tray under three-dimensional stress, the distortion energy density theory must be able to theoretically evaluate the ultimate strength, which is relatively reasonable, but the tensile strength of the GFRP bar under unidirectional tension cannot be used.

The change of thickness on the free surface of a variable thickness tray will cause stress concentration. In the future, the tray design should be optimized to eliminate the influence of adverse factors.

The content of glass fiber has a great impact on the strength of the GFRP bar. GFRP bar should be tested in field application and numerical simulation to ensure that the parameter value is reasonable.

By using the distortion energy density theory to analyze the internal force of the theoretical analysis and numerical simulation calculation, it can be concluded that the tensile stress of the inner wall of the free surface of the tray is the largest, which is the weakest part of the tray, and the above analysis conclusion is the same as the damage location of the tray measured in the nut-tray anchorage test. Therefore, the theoretical analysis, numerical simulation, and nut-tray anchorage test results are mutually verified, which further proves the rationality and feasibility of the theoretical analysis method.

## Figures and Tables

**Table 1:** Parameters of GFRP bars.

Diameter (mm)	Rib Height (mm)	Rib Width (mm)	Rib Spacing (mm)	Poisson’s Ratio	Density (g/cm^3^)
20	1.58	7.38	21.11	0.2	2.0

**Table 2:** Dimensions of nuts (Units in the table: mm).

Length	Big End Outside Diameter	Hexagon Flank Height	Hexagon Length	Cone Height
20	1.58	7.38	21.11	0.2
**Inside Diameter**	**Small End Outside Diameter**	**Conical Angle**	**Gap Length**	**Gap Width**
20	32.378	6	30	3.0

**Table 3:** Comparisons of theoretical analysis and numerical simulation.

Categories	Theoretical Analysis	Numerical Simulation
Elastic modulus	The tensile strength test of GFRP bars, 7.0 GPa.	
Poisson’s ratio	0.2	0.2
Friction coefficient between nut and inner wall	0.3	0.3
Tray thickness characteristics	Equal thickness	Variable thickness
Tray radius, (mm)	69.90	69.90
Tray center opening shape	Equivalent diameter round hole	Conical round hole
Boundary condition	Same as Section 2.3	
Load type and size	Equivalent uniform load, 6.4 MPa	
Analysis basis	According to Section 3.2., the deformation characteristics of the tray under maximum loading are as follows: the damage of the tray is the local cracking of the inner wall, and the deformation of the tray surface is a recoverable small deformation.	
Analysis method	Superposition of calculation results of thin plate bending and cavity expansion	Linear static analysis
Radial deflection characteristics of the tray	Theoretical calculation value of plate A > numerical simulation value > Theoretical calculation value of plate B, see Figure 9.	
Hoop stress characteristics of the loading surface of the tray	Controlled by hoop compressive stress, size: Theoretical calculation value of plate A > numerical simulation value ≈ Theoretical calculation value of plate B, see Figure 17.	
Radial stress characteristics of the free surface of the tray	Controlled by radial tensile stress, size: Theoretical calculation value of plate A > numerical simulation value ≈ Theoretical calculation value of plate B, see Figure 17.	
Radial stress of the inner wall of the tray, (MPa)	71.83 (plate B)	67.62 (mean value)
Hoop stress of inner wall of the tray, (MPa)	115.51 (plate B)	85.44 (mean value)
Maximum tensile stress of the free surface inner wall, (MPa)	207.50 (plate B)	190.98
Comparative conclusions	(1) The radial deflection, radial stress, and hoop stress calculated by equal thickness plate B are the same as the numerical simulation results. (2) The tensile stress of the inner wall of the free surface of the tray is the largest, which is the weakest part of the tray, and the above analysis conclusion is the same as the damage location of the tray measured in the nut-tray anchorage test.	

**Table 4:** Strength calculated values of the tray.

Position of Strength Calculation	Theoretical Calculation Value of Thin Plate B		Numerical Simulation Value of the Tray		
	Equivalent Inner Wall of Loading Surface	Inner Wall of Free Surface	Equivalent Inner Wall of Loading Surface	Inner Wall of Free Surface	Thickness Turning Area of Free Surface
σ_1_ (MPa)	138.09	−128.37	124.65	−146.44	−120.57
σ_2_ (MPa)	8.51	75.77	13.03	23.67	−26.81
σ_3_ (MPa)	−6.4	0	−6.4	0	0
σ_t_ (MPa)	171.74	207.50	154.21	190.98	140.18
σ_t_/σ_r_(%)	43.87	53.00	39.39	48.78	35.81

Note: Positive number represents tensile stress. Negative number represents compressive stress. σ_r_ is the standard tensile strength of GFRP bars, 391.5 MPa.

## Data Availability

No data, models, or code were generated or used during the study.
